# Patient expectations are better for immunotherapy than traditional chemotherapy for cancer

**DOI:** 10.1007/s00432-020-03336-1

**Published:** 2020-08-19

**Authors:** Andreas Ihrig, Jenniffer Richter, Carsten Grüllich, Leonidas Apostolidis, Peter Horak, Matthias Villalobos, Miriam Grapp, Hans-Christoph Friederich, Imad Maatouk

**Affiliations:** 1 Division of Psychooncology, Department of General Internal Medicine and Psychosomatic, University Hospital Heidelberg, Heidelberg, Germany; 2 Department of Medical Oncology, National Center for Tumor Diseases (NCT), University Hospital Heidelberg, Heidelberg, Germany; 3 Department of Translational Medical Oncology, National Center for Tumor Diseases (NCT), University Hospital Heidelberg and German Cancer Research Center (DKFZ), Heidelberg, Germany; 4 Department of Thoracic Oncology, University Hospital Heidelberg and Translational Lung Research Center Heidelberg (TLRC-H), Member of the German Center for Lung Research (DZL), Röntgenstr. 1, 69126 Heidelberg, Germany

**Keywords:** Immunotherapy, Chemotherapy, Knowledge, Expectations, Cancer patients, General population

## Abstract

**Purpose:**

The main aim of the study was to explore the expectations and knowledge of advanced-stage cancer patients about immunotherapy.

**Methods:**

This mixed methods study included 53 cancer patients on immune checkpoint inhibitors (ICIs), 55 cancer patients undergoing chemotherapy (CT), and 53 non-cancer patients. Participants’ expectations about ICIs and CT were compared. Additional qualitative data were derived from semi-structured interviews.

**Results:**

Among patients who did not receive ICIs, 63 (58%) had never heard of ICIs and 94 (87%) had large gaps in their knowledge of ICIs. Among ICI patients, 33 (62%) simply described ICIs without errors. ICI perception was positive, regardless of whether respondents received or had heard of ICIs, which became particularly evident when compared to CT. ICIs were rated as more promising, and all adverse effects were expected to be significantly lower than those of CT. Knowledge about ICIs was also limited in the interviewed ICI patients. Some patients reported adverse effects of ICIs that were mostly mild and well-tolerated or easily treated.

**Conclusions:**

The lack of understanding of ICIs should be improved by activities to increase the knowledge of ICI patients and the general population. In contrast to CT, ICIs invoked fewer negative associations with efficacy and toxicity. Therefore, attention should be paid to risk awareness when educating patients. (Clinical trial registration number: DRKS00011868)

**Trial Registration**: German clinical trials register, www.germanctr.de, number DRKS00011868.

## Introduction

Patients are assuming increasing responsibility in medical treatment decisions (Graffigna et al. 2012). In cancer treatment, patients’ knowledge and expectations are of considerable importance. Knowledge about and expectations of treatment are likely to affect patients’ decisions in dealing with daily issues during treatment and may affect quality of life (Henman et al. 2002). Negative expectations increase the risk of treatment-specific side-effects, nocebo side-effects, and non-adherence. Optimising individual expectations might be a promising strategy to improve side-effect burden, quality of life, and adherence during longer-term drug intake (Nestoriuc et al. 2016). Evidence suggests that the majority of cancer patients want to be fully informed about how their treatment works and what adverse effects could occur during treatment (Meredith et al. 1996). Several studies have investigated the comprehension of cancer patients and the general population. Prior research on conventional cancer therapy, such as chemotherapy (CT) has shown a high level of understanding in most of the general population, whereas knowledge about radiotherapy seems to be significantly lower (Chapman et al. 2003; Schnitzler et al. 2017). With regard to patients’ expectations of their prognosis, prior studies have shown that many patients with advanced cancer undergoing CT inaccurately perceive their illness as curable (Greer et al. 2014; Weeks et al. 2012). Studies focusing on patient perspectives’ of more recent cancer therapies, such as immunotherapy, are rare (Wong et al. 2019).

In the last decade, cancer immunotherapy has become another important pillar in cancer therapy (Adams et al. 2019; Drake et al. 2014). This change has resulted in ambiguity of the term immunotherapy in routine clinical practice, in which it is used as a collective term for cancer therapies. In this article, immunotherapy is used exclusively as a term for all cancer therapies based on the specific immune checkpoint inhibitors (ICIs) cytotoxic T-lymphocyte-associated protein 4 (CTLA-4) and programmed cell death protein 1/programmed death-ligand 1 (PD-1/PD-L1) (Emens et al. 2017). In some areas, ICIs have revolutionised cancer therapy in the last decade (Drake et al. 2014; Leprieur et al. 2017).

The expectations of treatment are complex and can be traced back to several sources of information (Devine et al. 2003). Reports on ICIs have increasingly appeared in the media. Some titles of books or magazines alone (e.g. *The Immunotherapy Revolution: The Best New Hope For Saving Cancer Patients’ Lives* (Williams 2019) or *Breakthrough of the year: Cancer Immunotherapy* (Couzin-Frankel 2013)) can raise high expectations. In 2018, the Nobel Prize was awarded to researchers for the discovery of immune checkpoint inhibition as cancer immunotherapy, which has brought ICIs further to the forefront (Guo 2018).

Clinical observations have shown that many patients are rather sceptical about CT because of a negative perception of toxic adverse effects, such as hair loss, nausea, and damage to the mucous membranes, and even a belief that ‘chemotherapy is poison’. Thus, even the names of chemotherapeutics are designed to counteract negative associations and achieve positive connotations (Abel and Glinert 2008). During therapy with ICIs, so-called immune-related adverse events (irAEs) may occur, in which the immune system can attack its own body structures (Haanen et al. 2017). Scientific studies addressing patients’ views on ICIs are rare.

From the patient perspective, the possession of knowledge is associated with a sense of control and, consequently, with reduced anxiety (Chapman et al. 2003; Henman et al. 2002). Furthermore, more knowledgeable patients are able have balanced discussions with their physician and are more likely to follow recommendations. In the case of ICIs, patient knowledge of irAEs is crucial for patient safety and treatment decision-making. A large proportion of patients experience adverse effects that are usually easily treated if they are detected early (Wang et al. 2019). If higher-grade adverse effects occur, there is a need for urgency; ‘once irAEs have developed, prompt work-up is required and action should be taken to prevent further aggravation’ (Haanen et al. 2017). However, only one qualitative study has investigated patients’ experiences with immunotherapy (Wong et al. 2019).

The main aim of our study was to explore the expectations and knowledge of advanced-stage cancer patients (with and without ICI treatment experience) using qualitative and quantitative approaches. Possible prejudices, biases, unrealistic expectations and misconceptions about ICIs were identified. This study offers physicians important guidance when discussing or educating cancer patients about immunotherapy.

## Methods

We performed a mixed methods study with the use of quantitative and qualitative research approaches to gain a deeper understanding of the research’s purpose.

### Quantitative evaluation

With the results of a survey, we compared patients’ knowledge about and expectations of ICI treatment among three different groups of patients. All patients were recruited from several departments of the National Center for Tumor Diseases and from other departments of the University Hospital Heidelberg. The general inclusion criteria were age ≥ 18 years, very good knowledge of the German language, and a written declaration of consent. The first group included advanced-stage cancer patients who already had experience making therapy decisions about ICIs. They were currently receiving or had completed palliative ICI treatment within the last year. The second group included advanced-stage cancer patients who were currently receiving or had completed palliative CT within the last year. Patients in the second group did not have any experience with immunotherapy. The third group comprised non-cancer (NoC) patients with non-palliative diseases other than cancer. The exclusion criteria for the third group were any close association with immunotherapy or CT, either professionally or personally, such as having close relatives who had experience with cancer therapy.

To achieve sufficient power (0.8) in a three-group ANOVA to detect medium size differences with α = 0.05, the sample size was determined to be at least 52 participants in each of the three groups (Cohen 1992). Between March 2017 and February 2018, 232 patients of the university hospital Heidelberg who met the inclusion criteria were asked to participate in the study. 71 patients did not want to participate due to participation in other studies, time reasons or without giving reasons. 161 (69%) patients agreed to participate.

Patients’ knowledge and expectations were measured using a 20-item questionnaire developed for this study. The items were partly adapted from other scales that measure the emotions or expectations of cancer patients (Ihrig et al. 2011; Mitchell et al. 2010). The questionnaire started with a question about if patients had ever heard about immunotherapy for cancer. The following open-ended question addressed knowledge about ICIs. The answers provided by the patients were noted by the interviewer, and patients’ knowledge was rated on a four-point scale from completely correct to completely false. Several closed-ended questions focused on further themes concerning ICIs and CT. There were seven questions comparing ICIs and CT (e.g. their costs, adverse effects and probability of success). A translation of the questionnaire is available upon request’.

### Qualitative evaluation

The qualitative data for the present study were derived from 12 semi-structured interviews with patients who were receiving ICI therapy or had received ICI therapy within the prior 12 months. The interviews were conducted between October 2017 and March 2018. Participants had to be physically able to follow the interview procedure. A topic guide was developed based on prior research, the literature and discussions with experienced oncologists and psychologists. Table 1 displays the main questions in the interview guide. All respondents consented to the interview being audiorecorded. The interviews were transcribed verbatim, and all data were managed and coded using qualitative data software (MAXQDA). Based on the typed notes, a summary content analysis according to (Mayring 2004) was performed by two independent researchers (JR and IM), which entailed discussing and reviewing the accuracy of the coding and defining a final set of categories and themes. We developed inductive categories for coding based on methods gathered from theory and the literature. Differences were discussed until a consensus was reached.

**Table 1:** Topics and corresponding questions from the interview guide (translated from German)

I. Introductory question
Please tell me what you associate with the term “immunotherapy” for cancer.
II. Mechanism/principle of action of immunotherapy in cancer
How do you think “immunotherapy” works for cancer?
What do “immunotherapies” for cancer do for the patient from your point of view?
What do you think is special about “immunotherapies” for cancer?
III. Side effects of immunotherapy in cancer
In your opinion, what side effects are possible with “immunotherapy”?
To what extent do you think these side effects might be relevant for cancer
patients?
IV. Comparison with other cancer therapies
If one compares “immunotherapy” for cancer in general with other cancer therapies:
To what extent do you think there might be differences to other cancer therapies?
To what extent do you think there might be similarities with other cancer therapies?
V. Cancer therapy
Which therapies have you received so far before the “immunotherapy” and how have you fared with these therapies?
You are currently receiving the “immunotherapeutic drug”
How did it come to the decision to carry out this therapy in particular?
How are you doing with the current therapy?
What expectations and hopes do you have regarding “immunotherapy”?
VI. Therapy clarification and decision
How did you feel about your education on “immunotherapy”?
What do you think: To what extent should you as a patient be involved in the decision for or against a therapy?
VII. Final question
Is there anything else you would like to note or add in conclusion?

### Statistical analyses

Absolute and relative frequencies were calculated for categorical data. Groups were compared with *χ*^2^ tests. For continuous variables, we present the mean ± standard deviation, median and range. We used an analysis of variance (ANOVA) or the *χ*^2^ test to compare variables among the groups and the paired *t* test to compare ratings. For the comparisons of the subgroups, we used an explorative mixed-ANOVA with a Greenhouse–Geisser correction. The post hoc tests Tukey HSD (honestly significant difference) for between-subjects and repeated measures ANOVA for within-subject factors were used. For all tests, *p *< 0.05 was considered to indicate statistical significance. For multiple comparisons we used the Bonferroni correction. All calculations were performed using SPSS 24.0 (IBM, Corp., Armonk, NY).

Informed consent was obtained from all participants. Ethical approval was received in May 2017 from the Ethics Committee of the Medical Faculty in Heidelberg (S-060/2017). The study was registered with the German Clinical Trials Register (DRKS00011868).

## Results

### Quantitative survey

Table 2 lists the demographic characteristics of the patients.

**Table 2:** Demographic characteristics of the patients

	All patients *N *= 161	ICI patients *N* = 53	CT patients *N* = 55	NoC patients *N* = 53	*p*
Gender					
Female	67 (42%)	21 (40%)	22 (40%)	24 (45%)	0.803
Male	94 (58%)	32 (60%)	33 (60%)	29 (55%)	
Age (years)*	58.5 ± 14.4 61 (18-89)	59.5 ± 11.7 60 (31–82)	62.4 ± 11.0 64 (31-79)	53.3 ± 18.1 54 (18-89)	0.003
Permanent partnership	113 (70%)	35 (66%)	39 (71%)	39 (74%)	0.690
Children	119 (74%)	43 (81%)	40 (73%)	36 (68%)	0.293
Education (school level)					
Elementary	71 (44%)	22 (42%)	23 (42%)	26 (49%)	0.342
Middle	46 (29%)	13 (25%)	15 (27%)	18 (34%)	
High	44 (827%)	18 (34%)	17 (31%)	9 (17%)	
Working status					
Employed	49 (30%)	12 (23%)	14 (25%)	23 (43%)	0.137
Retired	88 (55%)	32 (60%)	35 (64%)	21 (40%)	
Other	24 (15%)	9 (17%)	6 (10%)	9 (17%)	

*Mean ± standard deviation, median and range

There were more female than male participants, and NoC patients were younger than patients in the other groups. No significant differences were found among the groups for the other characteristics. Table 3 lists the primary underlying diseases of the 161 patients in the three study groups.

**Table 3:** The comorbidities of the patients in the three study groups

	ICI patients *N* = 53	CT patients *N* = 55	NoC patients *N* = 53
Dermatological tumours	23 (43%)	0 (0%)	–
Thoracic tumours	20 (38%)	21 (38%)	–
Gastrointestinal tumours	2 (4%)	27 (49%)	–
Other tumours	8 (15%)	7 (13%)	–
Cardiovascular diseases	–	–	26 (49%)
Gynaecological diseases	–	–	12 (23%)
Other non-oncological diseases	–	–	15 (28%)
Metastasis	51 (96%)	47 (85%)	–
Experience with CT	26 (49%)	55 (100%)	0 (0%)
ICI therapy			
PD-1 inhibitors	43 (81%)	–	–
Other	10 (19%)	–	–

The ICI patients mainly had dermatological or thoracic tumours, whereas the CT patients mainly had gastrointestinal or thoracic tumours. The NoC patients mainly had cardiovascular or non-malignant gynaecological diseases. The answers to the knowledge questions about ICIs are shown in Table 4.

**Table 4:** Answers to knowledge questions about immune checkpoint inhibitors

	All patients *N* = 161	ICI patients *N* = 53	CT patients *N* = 55	NoC patients *N* = 53	*χ*^2^-test (*p*)
Have you ever heard the term “Immunotherapy” in connection with the treatment of cancer?					
Yes	98 (61%)	53 (100%)	27 (49%)	18 (34%)	**< 0.001**
No	63 (39%)	0 (0%)	28 (51%)	35 (66%)	
Classification of the state of knowledge					
Correct	28 (17%)	23 (43%)	4 (7%)	1 (2%)	**< 0.001**
Mainly correct, but with incomplete information	19 (12%)	10 (19%)	7 (13%)	2 (4%)	
Partially incorrect information	30 (19%)	7 (13%)	14 (25%)	9 (17%)	
Incorrect or no specification made	84 (52%)	13 (25%)	30 (55%)	41 (77%)	

In the initial question, 98 (61%) patients stated they had heard of ICIs in connection with cancer treatment, while 63 (39%) stated they had not. To investigate the possible effects of prior knowledge, we statistically compared all assessments between these two groups. As these calculations did not reveal any significant differences (*p* = 0.10–0.90), we did not present them in the results section.

Most patients (114; 71%) could not correctly describe the mechanism of action of ICIs. While 33 (62%) ICI patients were able to simply describe ICIs without errors, significantly fewer CT patients (20%) and NoC patients (6%) were able to do so. When asked if they remembered when they first heard about ICTs, 74 patients responded 1.3 ± 1.2 (1.1; 0–5.8) years prior. Regarding CT, 26 patients initially stated that they had always known this term, and 81 patients remembered having first heard the term 28.6 ± 14.2 (30.4; 1.3–55.4) years prior.

Table 5 shows a comparison of the ratings of ICI and CT by the 161 participants. Participants had to rate each aspect on a scale from 0 (not at all, very low) to 10 (very much, very high).

**Table 5:** Comparison of the mean ± standard deviation of the ratings of immune checkpoint inhibitors and chemotherapy by 161 patients

	Immunotherapy	Chemotherapy	Paired *t* test (*p*)^a^
Costs	7.4 ± 2.2	6.9 ± 2.0	0.021
Time required	5.6 ± 2.1	6.9 ± 1.8	**< 0.001**
Prospect of success	7.3 ± 1.9	6.5 ± 2.1	**< 0.001**
Minor adverse effects	4.7 ± 2.5	7.7 ± 2.0	**< 0.001**
Severe adverse effects	4.0 ± 2.2	7.1 ± 2.4	**< 0.001**
Life-threatening adverse effects	2.7 ± 2.3	4.6 ± 2.9	**< 0.001**
This is the future of cancer therapy	7.3 ± 2.3	5.4 ± 2.6	**< 0.001**

^a^*p* < 0.007 is defined as significant (in bold) based on Bonferroni correction for multiple comparisons

The participants made a significantly different assessment of ICIs and CT in each area except costs. ICIs were considered to require less time, have a better prospect of success, and have less adverse effects than CT. In addition, ICIs were more often stated to be the future of cancer therapy than CT.

Table 6 shows the comparison of the ratings of ICIs and CT by the patient groups.

**Table 6:** Comparisons of the ratings of ICI and CT in the three patient groups

	ICI patients (*N* = 53)		CT patients (*N* = 55)		NoC patients (*N* = 53)		Mixed ANOVA^a^
	ICI	CT	ICI	CT	ICI	CT	
Costs	8.3 ± 1.9*^,+^	6.3 ± 1.9	7.3 ± 2.2	7.6 ± 1.9	6.5 ± 2.3	6.8 ± 2.0	F (2, 154) = 14.875, ***p* < 0.001**
Time required	5.2 ± 2.2	6.5 ± 2.0	6.0 ± 2.1	7.1 ± 1.7	5.6 ± 1.9	7.1 ± 1.5	F (2, 155) = 0.418, *p* = 0.659
Prospect of success	8.3 ± 1.8*^,+^	6.1 ± 2.4	7.1 ± 1.8	7.5 ± 1.7^+^	6.4 ± 1.7*	5.8 ± 1.7	F (2, 153) = 16.091, ***p* < 0.001**
Minor adverse effects	4.5 ± 3.1	7.5 ± 1.9	5.0 ± 2.3	7.2 ± 2.3	4.5 ± 1.9	8.3 ± 1.5	F (2, 155) = 4.701, *p* < 0.010
Severe adverse effects	3.4 ± 2.6*	6.9 ± 2.4	4.4 ± 2.1*	6.4 ± 2.7	4.2 ± 2.0*	8.1 ± 1.6^+^	F (2, 155) = 6.906, ***p* = 0.001**
Life-threatening adverse effects	2.3 ± 2.6*	5.1 ± 3.0	2.8 ± 2.2*	3.9 ± 2.8	2.9 ± 2.0*	4.9 ± 2.7	F (2, 155) = 6.167, ***p* = 0.003**
This is the future of cancer therapy	8.3 ± 1.7	5.6 ± 2.8	7.2 ± 2.5	5.9 ± 2.6	6.4 ± 2.2	4.7 ± 2.3	F (2, 154) = 2.556, *p* = 0.081

^+^Rating differs significantly from the two ratings of the other patient groups (post hoc between-subjects, Tukey HSD)

*ICI rating differs significantly from the CT rating (post hoc within-subject, repeated measures ANOVA)

^a^*p* < 0.007 is defined as significant (in bold) based on Bonferroni correction for multiple comparisons

In the mixed-ANOVA there were statistically significant interactions in costs, prospect of success, and severe and life-threatening adverse effects between rating of ICI and CT and the patient groups. According to the between-subjects post hoc analyses ICI patients rated the costs of their own therapy higher (1.012, *p *= 0.041; 1.837, *p *< 0.001) and the prospect of success more promising (1.250, *p *= 0.001; 1.981, *p *< 0.001) than the CT and NoC patients. CT patients rated the prospect of success of their own therapy higher than ICI patients (1.346, *p *= 0.002) and NoC patients (1.673, *p *< 0.001). NoC patients rated severe adverse effects of CT higher than ICI patients (1.247, *p *= 0.016) and CT patients (1.624, *p *= 0.001). Life-threatening adverse effects of ICI were simultaneously rated lower than CT in all groups. In the within-subject post hoc analyses, all ICI patients assessed ICIs to be better than CT. CT patients’ estimates of the costs and prospect of success of ICIs and CT did not significantly differ. NoC patients’ estimates of the cost of ICIs and CT did not significantly differ, while their assessments of the prospect of success and adverse effects favoured ICIs.

### Qualitative content analysis

Interviews were conducted with 12 palliative patients (4 women, 8 men) in stable conditions who were receiving ICI therapy or had received ICI therapy within the previous 12 months. On average, the interviews lasted 32 min, with a range from 16–65 min. The characteristics of the participants in the qualitative part are shown in Table 7.

**Table 7:** Characteristics of the participants in the qualitative interview

	ICI patients *N* = 12
Age (years), mean ± SD (Mitchell et al.)	61.5 ± 11.7 (40–83)
Type of cancer	
Malignant melanoma	3 (25%)
NSCLC	2 (17%)
Urogenital cancer	2 (17%)
Head and neck cancer	3 (25%)
Colon cancer	1 (8%)
Malignant germ cell tumour	1 (8%)
Metastasis	12 (100%)
Monotherapy with PD-1 inhibitor	
Nivolumab	8 (67%)
Pembrolizumab	4 (33%)
Time since first administration of ICI (months), mean ± SD (Mitchell et al.)	12.7 ± 9.2 (1–27)

### Interviews

Patients who had received ICI therapy named several topics, from which four main categories were derived: prognosis, quality of life, adverse effects and decision-making about therapy. The following section briefly describes the main categories.

### Prognosis

‘I hope ICI helps me. I hope it helps well. And I am optimistic that it will succeed.’

The interviews showed that patients mainly hoped for stable disease or tumour regression from ICI therapy. This idea was usually communicated to the patients by the attending physicians. In particular, the interviewees hoped to gain more years with quality of life. Overall, the affected patients showed both an accurate prognostic awareness of their unfavourable prognosis and a simultaneous, albeit less pronounced, hope of recovery.

### Quality of life

‘The therapy doesn’t handicap me and doesn’t cause any difficulties, health or mental or whatever.’

A recurring motif in the patient interviews was the hope of participating in everyday life. Overall, the patients wished to lead a normal life during ICI therapy. Nevertheless, it was reported that the everyday life of the patients had to be adapted to ICI therapy, the time required for tasks or the need for breaks, since the therapy affects the body to a certain extent. However, the interview responses suggested that ICI therapy could integrate well into patients’ everyday lives (i.e. through outpatient treatment and few infusions). In addition, the comparisons frequently made by patients with CT also played a role, with patients retrospectively reporting poorer quality of life.

### Adverse effects

‘Well, I’ve actually had no side effects so far, I don’t think so at all… Or itching after all. It could come from this therapy, because it becomes normal again after three or 4 days. I don’t know if it really is like that, but I am more and more convinced that it comes from ICI.’

Some of the interviewees reported adverse effects that they had personally experienced. The patients’ comments suggested that adverse effects integrated well into daily life or could be easily treated. Severe or limiting adverse effects were temporary. The respondents stated that they experienced fewer adverse effects with ICI therapy and found ICI to be less restrictive than CT. This comparison was based on their own experiences with previous oncological therapies or the experiences of friends and acquaintances. Well-tolerated CT was rarely reported. In the course of the interviews, patients were able to name several adverse effects of ICI therapy. In some interviews, the occurrence of adverse effects was interpreted as a sign of therapy response, or at least discussed as such.

### Treatment decisions

‘I didn’t decide. I think that was Dr X. She was the first one to ask me if we could try. And then I said: “Yes, it’s okay for me. I’ll try it.”’

The interviewees reported that the recommendation for ICI therapy was made by the majority of the attending physicians. Previous knowledge through media reports was rare. In most cases, the decision of therapy was preceded by tumour progression or a failure to respond to the previous oncological therapy. The interviewees also stated a lack of an alternative therapy if a rapid remission was needed or highly advanced metastatic disease could also lead to ICI therapy. First-line therapy approval was less frequent. Overall, the interviewees stated that they wanted to make the final decision for or against therapy themselves after the corresponding recommendation by the physician. The interviews showed that in some cases, patients were more reluctant to concur with CT than with ICIs. Patients also focused on communication with and care provided by their clinicians. In this respect, the respondents were highly satisfied and grateful.

## Discussion

### ICI perception

The main result of our study was that ICI perception was very positive. This finding is consistent with the findings of an interview study that evaluated the experiences of 23 patients with advanced melanoma that were receiving immunotherapy (Wong et al. 2019). Those patients described perceived hope and high expectations of efficacy with ICIs.

Surprisingly, the positive ratings of ICI were stable, regardless of whether respondents received ICIs or whether they had never heard of ICIs. This positive connotation became particularly evident when ICIs were compared to CT. Unlike CT, the term immunotherapy has not been used for decades. There are obviously few other associations with ICIs than the ‘ICI is a chance’ reports in the press, the connection with the awarding of the Nobel Prize, and the information from physicians treating patients.

The term CT belongs to the general vocabulary learned in childhood, at least in Western culture. In addition to frequent critical media reports, films such as *Dying Young*, in which one of the main actors calls CT poison, or Solzhenitsyn’s novel *Cancer Ward*, in which cancer was linked with death and hopelessness, that shape the image of CT in the population (Clark 1999; Kaptein and Lyons 2010). However, CT has recently been described in films and television shows (e.g. *Bucket List* and *Breaking Bad*) as compatible with everyday activities. CT is further associated with adverse effects such as vomiting and hair loss. It is unique in that CT is a treatment that is itself a source of suffering (Bell 2009). For cancer patients, the prospect of CT is filled with fear and terror (Bell 2009; Cowley et al. 2000; Hofman et al. 2004; Sohl et al. 2009). Our results, however, show that these types of associations do not exist for ICIs.

One aspect that could contribute to this difference is that the word CT (and the word radiotherapy) names the subject of treatment. Chemicals and radiation are external influences that are harmful to humans in other contexts (e.g. poison and nuclear weapons). In contrast, the core of the word immunotherapy is associated with a positive idea of being immune and a sense of a protective mechanism. This association could contribute to a positive expectation towards immunotherapy; in particular, it could partly explain the expectations of people who have never heard of it.

### Experiences with ICIs

In a review of 125 studies, many adverse effects of ICIs were described (Wang et al. 2019). Among more than 18,000 patients, 66% experienced at least one adverse event, and 14% experienced at least one adverse event with high severity (Wang et al. 2019). In contrast, our ICI patients described their therapy to have relatively few adverse effects. Their positive assessment of ICIs seems to be confirmed by their positive experiences. In a study by Wong et al., 23 patients with advanced melanoma who were interviewed also perceived immunotherapy to be extremely safe (Wong et al. 2019). These results indicate that outpatient ICI therapy can generally integrate well into daily life.

### ICI knowledge

The knowledge about ICIs in the patients in our survey was deficient. It can be assumed that the general population’s knowledge about ICIs and their possible adverse effects is also deficient. ICI patients are better informed than others, but the gaps in their knowledge about the possible adverse effects of ICIs remain. Similar to our findings, the findings of Wong et al. in their study with 23 patients demonstrated that the patients were unable to provide specific knowledge about ICIs, such as potential toxicities or details on likely success rates (Wong et al. 2019). These gaps in knowledge can become dangerous if patients overlook early symptoms and react too late. Adverse effects from ICIs must be treated as early as possible; otherwise, they become life-threatening (Collins et al. 2017; Friedman et al. 2016; Hottinger 2016).

### Implications for physicians and medical education

Any oncologist who informs patients about CT is aware of the fears of most patients regarding this treatment. The different expectations of patients with ICIs should, therefore, lead to a different focus of patient information and counselling. With these high expectations of ICIs, clinicians face the challenge of providing realistic and balanced advice (Wong et al. 2019). While CT often has false-negative expectations that need to be invalidated, ICIs are more likely to be associated with misconceptions about their efficacy and safety. Therefore, ICIs require more focus on risk awareness than CT, in which some patients are more likely to be convinced to undergo therapy by physicians. A lack of knowledge about adverse effects in ICI patients could lead to an underestimation of the risk of and possibly to neglect important symptoms. Patients are willing to actively participate in adverse effect management, but they need correct and adapted instruction from their practitioners (Schwappach and Wernli 2010). It is important to identify and report symptoms to treat them early and avoid life-threatening conditions. Therefore, special attention should be paid to this aspect when educating patients, even if physicians tend to emphasise positive aspects and run the risk of being rated less favourably by patients if they provide negative information (Bousquet et al. 2015; Weeks et al. 2012).

The possible adverse effects of ICIs were also estimated to be relatively harmless in the NoC group. The assessments of this group can be used as a guideline for the expectations of relatives of cancer patients. Accordingly, relatives of cancer patients also risk underestimating possible early symptoms and may not be able to help patients take the right actions.

## Limitations

One limitation of our study is that our patients did not comprise a representative sample. Nevertheless, with a sample size of 161, we believe that we interviewed a sufficiently large group to be able to represent general trends.

Furthermore, immunotherapy is a rapidly changing field. On average, the term immunotherapy was only known to the participants for a little more than a year. It can be assumed that with increasing experience and broad implementation of ICIs in routine cancer care, expectations towards ICIs will shift in the coming years. In addition to previous results, our results may, therefore, serve as a basis for comparison for later considerations.

## Conclusions

The lack of understanding about ICIs should be improved by activities to increase the knowledge of not only ICI patients, but also the general population.

In contrast to CT, ICIs currently invoke fewer negative associations with regard to efficacy and toxicity. Therefore, special attention should be paid on risk awareness when educating patients.

As ICIs become an established part of routine clinical practice, it should be observed whether the expectations of ICIs change.

## Abbreviations

ANOVA: Analysis of variance
CT: Chemotherapy
HSD: Honestly significant difference
ICI: Immune checkpoint inhibitor
irAE: Immune-related adverse event
NoC: Non-cancer

## References

[CR1] Abel GA, Glinert LH (2008). Chemotherapy as language: sound symbolism in cancer medication names. Soc Sci Med.

[CR2] Adams S (2019). Current landscape of immunotherapy in breast cancer: a review. JAMA Oncol.

[CR3] Bell K (2009). ‘If it almost kills you that means it’s working!’Cultural models of chemotherapy expressed in a cancer support group. Soc Sci Med.

[CR4] Bousquet G, Orri M, Winterman S, Brugière C, Verneuil L, Revah-Levy A (2015). Breaking bad news in oncology: a metasynthesis. J Clin Oncol.

[CR5] Chapman K, Abraham C, Jenkins V, Fallowfield L (2003). Lay understanding of terms used in cancer consultations. Psycho-Oncology.

[CR6] Clark RA (1999). Reel oncology: how Hollywood films portray cancer. Cancer Control.

[CR7] Cohen J (1992). A power primer. Psychol Bull.

[CR8] Collins LK, Chapman MS, Carter JB, Samie FH (2017). Cutaneous adverse effects of the immune checkpoint inhibitors. Curr Probl Cancer.

[CR9] Couzin-Frankel J (2013). Breakthrough of the year 2013: cancer Immunotherapy. Cancer Immunother Sci.

[CR10] Cowley L, Heyman B, Stanton M, Milner SJ (2000). How women receiving adjuvant chemotherapy for breast cancer cope with their treatment: a risk management perspective. J Adv Nurs.

[CR11] Devine D, Parker PA, Fouladi RT, Cohen L (2003). The association between social support, intrusive thoughts, avoidance, and adjustment following an experimental cancer treatment. Psycho-Oncol.

[CR12] Drake CG, Lipson EJ, Brahmer JR (2014). Breathing new life into immunotherapy: review of melanoma, lung and kidney cancer. Nat Rev Clin Oncol.

[CR13] Emens LA (2017). Cancer immunotherapy: opportunities and challenges in the rapidly evolving clinical landscape. Eur J Cancer.

[CR14] Friedman CF, Proverbs-Singh TA, Postow MA (2016). Treatment of the immune-related adverse effects of immune checkpoint inhibitors: a review. JAMA Oncol.

[CR15] Graffigna G, Libreri C, Bosio C (2012). Online exchanges among cancer patients and caregivers: constructing and sharing health knowledge about time. Qualit Res Organiz Manag.

[CR16] Greer JA (2014). Perceptions of health status and survival in patients with metastatic lung cancer. J Pain Symptom Manage.

[CR32] Guo ZS (2018). The 2018 Nobel Prize in medicine goes to cancer immunotherapy. BMC Cancer.

[CR17] Haanen J, Carbonnel F, Robert C, Kerr K, Peters S, Larkin J, Jordan K (2017). Management of toxicities from immunotherapy: ESMO clinical practice Guidelines for diagnosis, treatment and follow-up. Ann Oncol.

[CR18] Henman M, Butow P, Brown R, Boyle F, Tattersall M (2002). Lay constructions of decision-making in cancer. Psycho-Oncol.

[CR19] Hofman M (2004). Cancer patients’ expectations of experiencing treatment-related side effects: a University of Rochester Cancer Center-Community Clinical Oncology Program study of 938 patients from community practices. Cancer.

[CR20] Hottinger AF (2016). Neurologic complications of immune checkpoint inhibitors. Curr Opin Neurol.

[CR21] Ihrig A (2011). Treatment decision-making in localized prostate cancer: why patients chose either radical prostatectomy or external beam radiation therapy. BJU Int.

[CR22] Kaptein AA, Lyons AC (2010). Cancer ward: patient perceptions in oncology. J Health Psychol.

[CR23] Lange M (2014). Cognitive dysfunctions in elderly cancer patients: a new challenge for oncologists. Cancer Treat Rev.

[CR24] Leprieur EG, Dumenil C, Julie C, Giraud V, Dumoulin J, Labrune S, Chinet T (2017). Immunotherapy revolutionises non-small-cell lung cancer therapy: results, perspectives and new challenges. Eur J Cancer.

[CR25] Mayring P, Flick U (2004). Qualitative content analysis. A companion to qualitative research.

[CR26] Meredith C, Symonds P, Webster L, Lamont D, Pyper E, Gillis CR, Fallowfield L (1996). Information needs of cancer patients in west Scotland: cross sectional survey of patients’ views. BMJ.

[CR27] Mitchell AJ, Baker-Glenn EA, Granger L, Symonds P (2010). Can the distress thermometer be improved by additional mood domains? Part I. Initial validation of the emotion thermometers tool. Psycho-Oncology.

[CR28] Nestoriuc Y, von Blanckenburg P, Schuricht F, Barsky A, Hadji P, Albert U-S, Rief W (2016). Is it best to expect the worst? Influence of patients’ side-effect expectations on endocrine treatment outcome in a 2-year prospective clinical cohort study. Ann Oncol.

[CR29] Schnitzler L (2017). Communication during radiation therapy education sessions: the role of medical jargon and emotional support in clarifying patient confusion. Patient Educ Couns.

[CR30] Schwappach DL, Wernli M (2010). Am I (un) safe here? Chemotherapy patients’ perspectives towards engaging in their safety. Qual Saf Health Care.

[CR31] Sohl SJ, Schnur JB, Montgomery GH (2009). A meta-analysis of the relationship between response expectancies and cancer treatment-related side effects. J Pain Symptom Manage.

[CR33] Wang Y (2019). Treatment-related adverse events of PD-1 and PD-L1 inhibitors in clinical trials: a systematic review and meta-analysis. JAMA Oncol.

[CR34] Weeks JC, Catalano PJ, Cronin A, Finkelman MD, Mack JW, Keating NL, Schrag D (2012). Patients’ expectations about effects of chemotherapy for advanced cancer. N Engl J Med.

[CR35] Williams JR (2019). The immunotherapy revolution: the best new hope for saving cancer patients’ lives.

[CR36] Wong A, Billett A, Milne D (2019). Balancing the hype with reality: what do patients with advanced melanoma consider when making the decision to have immunotherapy?. Oncologist.

